# Protocol for a double-blind, randomized controlled trial on the dose-related efficacy of omalizumab in multi-food oral immunotherapy

**DOI:** 10.1186/s13223-020-00419-z

**Published:** 2020-04-17

**Authors:** Alexandra Langlois, Marie-Hélène Lavergne, Hélène Leroux, Kerstin Killer, Pauline Azzano, Louis Paradis, Kathryn Samaan, Jonathan Lacombe-Barrios, Benoît Mâsse, Anne Des Roches, Philippe Bégin

**Affiliations:** 1 Department of Allergy and Immunology, Centre Hospitalier Universitaire Sainte-Justine, Montreal, QC Canada; 2 Department of Allergy and Immunology, Centre Hospitalier de l’Université de Montréal, 3175 Chemin de la Côte Sainte-Catherine, Montreal, QC H3T1C5 Canada; 3 Centre Hospitalier Sainte-Justine Research Center, Montreal, QC Canada; 4 School of Public Health, Université de Montréal, Montreal, QC Canada

**Keywords:** Food allergy, Oral immunotherapy, Desensitization, Omalizumab, Anti-IgE, Safety, Efficacy, Randomized controlled trial

## Abstract

**Background:**

Previous proof-of-concept studies have shown that a short course of omalizumab can safely accelerate the oral immunotherapy schedule for multiple allergens simultaneously. Considering the high cost of medication, the dose-related efficacy of omalizumab at decreasing the duration of oral immunotherapy up-dosing phase must be objectively quantified before cost–benefit analyses can be performed. The primary objective of this trial will be to compare the efficacy of 2 omalizumab dosages to placebo at decreasing time-to-maintenance dose during a symptom-driven multi-food OIT protocol.

**Methods:**

A total of 90 participants aged 6 to 25 with multiple food allergies (3 or more) will be enrolled at four sites in Canada. Participants will be randomized to: (A) Omalizumab 8 mg/kg per month (n = 36); (B) Omalizumab 16 mg/kg per month (n = 36); or (C) Placebo (n = 18). Study drug will be administered at full dosage for 12 weeks, then progressively tapered at 50% dosage (8 mg/kg vs 4 mg/kg vs placebo) for 4 weeks and at 25% dosage (4 mg/kg vs 2 mg/kg vs placebo) for another 4 weeks. After a pre-treatment period of 8 weeks, participants will undergo an initial food escalation (IFE) to an OIT mix containing 3 allergens and start daily home dosing with biweekly increases until a target daily maintenance of 1500 mg protein is achieved. The amount escalated at each visit will vary based on treatment tolerance according to a standardized up-dosing algorithm. Participants will be followed for at least 12 months following the initial food escalation. The primary endpoint will be time from IFE to the target maintenance dose of 1500 mg protein. Time-to-event analytic methods, including the log-rank test, will be used to compare the 3 arms.

**Discussion:**

This trial uses a novel pragmatic approach to compare OIT with omalizumab to OIT without omalizumab in a blinded manner, which allows to single out the effect of this anti-IgE medication on treatment effectiveness speed without the recourse to predetermined schedules. The innovative patient-centered up-dosing algorithm allows to maximise treatment effectiveness speed without compromising patient safety, regardless of whether the patient is on omalizumab or not. This study will also provide novel prospective data to inform on the optimal and most cost-effective dosage for this indication.

*Trial registration* ClinicalTrials.gov, NCT04045301, Registered 5 August 2019, https://clinicaltrials.gov/ct2/show/NCT04045301

## Background

Over the last decade, there has been a growing interest in the use of oral immunotherapy (OIT) to desensitize patients with food allergies [1, 2]. A recent systematic review has shown this approach to be effective and to be associated with an 80% improvement in quality of life [2–4].

While it can often be performed with relatively low amounts of resources and relative ease in patients with a mild allergy to a single food [5], it is usually not the case for those with severe and multiple food allergies. In these patients, home-dosing reactions and anaphylaxis are more likely, often requiring a prolonged up-dosing schedule with continuous patient support and intense safety monitoring. Frequent reactions can lead to family exhaustion and a decrease in quality of life [6, 7]. Due to legitimate safety, cost-effectiveness and logistical concerns, allergists currently offering OIT in clinic mostly focus on cases with a single food allergy. This is paradoxical as multiple food allergies (30% of cases [8–10]) are generally more severe, have a greater impact on quality of life and are less prone to resolve over time spontaneously [11–14]. Limited access to specialized clinics to administer extended treatments remains a barrier. One avenue that has been proposed is the combination of a short course of omalizumab with multi-food OIT to allow a rapid and safe desensitization.

### Use of omalizumab in oral immunotherapy

**Fig. 1:**
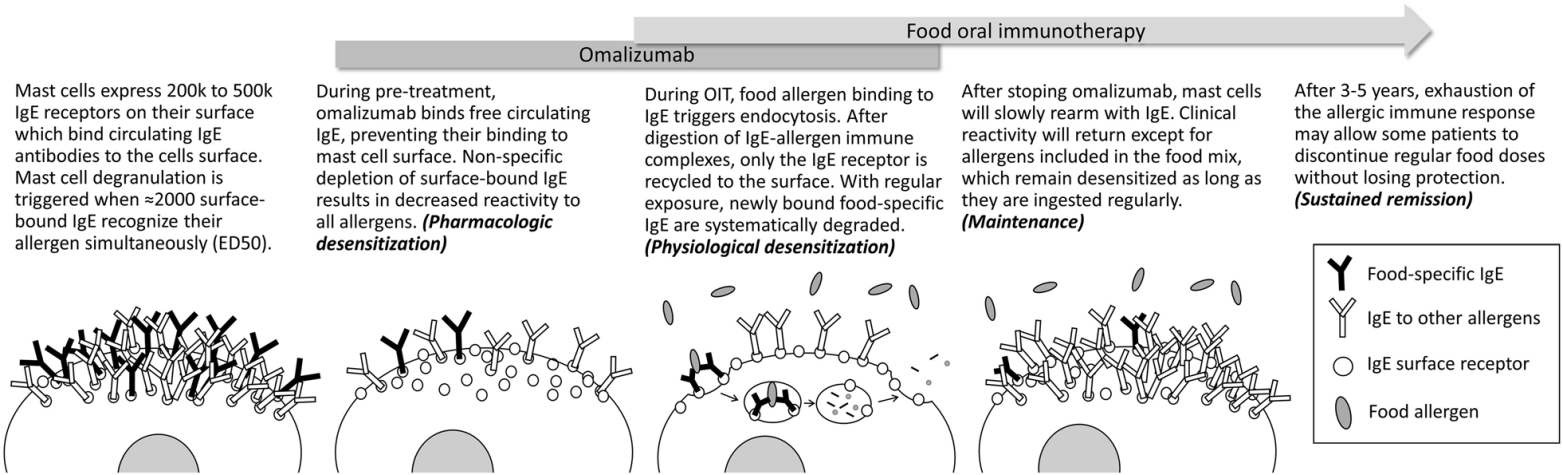
Conceptual model of omalizumab-enabled immunotherapy. ED50: Eliciting does triggering 50% of degranulation; IgE: immunoglobulin E; OIT: oral immunotherapy

Omalizumab is an anti-IgE monoclonal antibody, currently approved for asthma and chronic urticaria, which has been shown to drastically raise tolerance threshold to food allergens [15–17]. When used as adjunct to OIT, a short course of omalizumab can enable a rapid and safe escalation of food doses (Fig. 1) [18, 19]. Omalizumab binds free circulating IgE on its Fcε3 domain and impairs its binding to the high-affinity IgE receptor (FcεRI) on basophils or mast cells [20–22]. At therapeutic doses, it has also been shown to actively dissociate bound specific IgE from their receptor on the mast cell [23]. Another potential mechanism that has been proposed to reduce the risk of anaphylaxis is the direct neutralization of allergens in the blood stream by omalizumab-IgE complexes, serving as competitive inhibitors sweeping the allergen molecules entering the bloodstream before they can reach mast cells and basophils [24].

The use of omalizumab as adjunct therapy to OIT has been reviewed previously [25–28]. There are 6 uncontrolled trials to date currently indexed in MEDLINE conducted for milk (n = 26) [29–31], peanut (n = 39) [32, 33], egg (n = 12) [30, 34] and multiple food allergens (n = 25) [19]. All conclude to the safety of using omalizumab to achieve rapid desensitization with success rates of reaching maintenance greater than 93%. There are 3 proof-of-concept phase 2a trials investigating the use of omalizumab as adjunct to OIT:

- Wood et al. (n = 57; 1:1) compared omalizumab to placebo during a slow milk OIT schedule and found rates of sustained unresponsiveness (48% vs 36%) and desensitization (89% vs 71%) to be comparable at 2 years, demonstrating the futility of adding omalizumab to slow OIT (although it did effectively suppress systemic reactions to OIT) [35].
- MacGinnitie et al. (n = 37; 3.5:1) tested omalizumab as an adjunct to an accelerated peanut OIT schedule. At 14 weeks, 79% could tolerate 2 g of peanut proteins, compared to 12% in the placebo group (RR = 6.6), with 7% and 75% protocol failures (RR = 0.09), respectively [36].
- Andorf et al. (n = 48; 3:1) also tested omalizumab to placebo as adjunct to an accelerated schedule of multi-food OIT. At 28 weeks, 83% vs 33% could tolerate 2 g proteins of at least 2 foods (RR = 2.5). At 8 weeks, there were 8% vs 67% treatment failures in each group, respectively (RR = 0.12) [37].

**Fig. 2:**
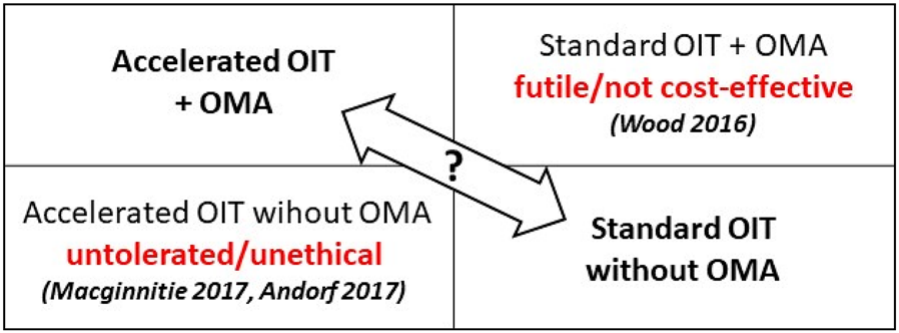
The clinical equipoise. OMA: omalizumab; OIT: oral immunotherapy

These trials readily show that *accelerated OIT is not tolerated without omalizumab*. This said, the benefit of omalizumab-enabled accelerated OIT (OEAOIT) has yet to be demonstrated over standard OIT, which is the option currently available in clinic (albeit not on a large scale) and therefore the right comparator (Fig. 2). Both approaches have been shown to be effective at inducing desensitisation over time, and to be safe. The main advantage of omalizumab in clinic would be to significantly reduce the length of the up-dosing phase, which is the most labor-intensive and resource-consuming part of treatment which contributes most to limiting access. The optimal dose to be used for this indication has also never been determined as all previous trials used the asthma dosage chart based on patient weight and total IgE. This is particularly relevant considering the high cost of medication, as lower dosages could make the difference for public coverage and universal access to treatment.

The objective of this trial is therefore to investigate the dose-related efficacy of omalizumab at decreasing the time to maintenance during OIT in participants 6 to 25 years old with multiple IgE-mediated food allergies.

## Method/design

### Study design

**Fig. 3:**
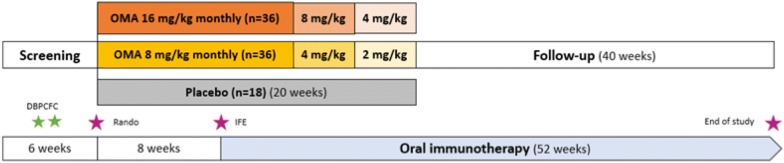
Overall study design. DBPCFC: double-blind placebo-controlled food challenge; IFE: initial food escalation; OMA: omalizumab

The study is a multi-center phase 2b clinical trial and will be conducted in a randomized controlled fashion comparing two dosages of omalizumab to placebo during a symptom-driven multi-food oral immunotherapy (OIT) protocol (Fig. 3). The study will be conducted in five research centers in Canada. This list will be updated on the clinical trial registration site throughout the trial (NCT04045301). Ninety participants will be recruited and randomized 2:2:1 to receive 20 weeks of omalizumab at monthly dosages of 16 mg/kg, 8 mg/kg or placebo. The study drug will be given at full dosage for a total of 12 weeks with a progressive taper during the last 8 weeks. Multi-food OIT will be started after a pre-treatment period of 8 weeks (Table 1). It will be performed with biweekly up-dosing according to a symptom-driven schedule until the target dose of 1500 mg of food protein is reached (500 mg per food). All participants will be consented by site investigators in accordance with the Declaration of Helsinki. A separate consent will be presented for biobanking purposes as per site policies. The study has been approved by the coordinating centers’ Research Ethics Committees and is registered in Clinicaltrials.gov (NCT04045301). Ethics approval will be obtained at each study site prior to initiation. The study will be conducted according to Good Clinical Practices (GCP) and all participating sites will need to provide certifications of GCP/Division 5 training for all those involved in the conduct of the study.

**Table 1:** Schedule of procedures

	Screening			Study drug pre-treatment phase				Study drug + OIT up-dosing phase						OIT up-dosing without study drug phase^a^							Early term	Unsched visit
Visits	V1	V2	V3	V4	V5^j^	V6	V7^j^	V8	V9	V10	V11	V12	V13	V14	v15 + …	V20	v22 + …	PC36^n^		EoS	ET	UV^m^
Visit week	Scr			W-8	W-6	W-4	W-2	D1	W2	W4	W6	W8	W10	W12	q2w	W24	q2w	W36		W52+		
Time window		No more than 7 days apart		Max 90 days after V1	±4 days			±4 days					^e^	±7 days	^e^	±7days	^e^	±7 days		±7 days		
Informed consent/assent	X																					
Check eligibility criteria	X			X																		
QoL questionnaire*	X													X		X				X	X	
Treatment-related cost evaluation questionnaire**	X	X	X	X	X	X	X	X	X	X	X	X	X	X	X	X	X	X		X	X	X
Demographics	X																					
Medical history	X																					
Allergy history	X																					
Weight and height^b^	X	X	X	X				X		X^b^		X^b^		X	X^b^	X	X^b^			X	X	
Vital signs	X	X^h^	X^h^					X^h^														
Complete Physical Examination	X																			X	X	
Brief Physical Examination		X^i^	X^i^	X	X	X	X	X^i^	X	X	X	X	X	X	X	X	X					X
Skin Prick Test	X							X						X		X				X	X	
Blood sampling	X							X		X^o^				X		X				X	X	
Optional stool sample collection		X^p^						X^q^						X						X	X	
Urine pregnancy test^c^	X			X				X												X	X	
Spirometry	X																					
Peak expiratory flow (PEF)	X	X	X					X														
Prior/concomitant medication^d^	X	X	X	X	X	X	X	X	X	X	X	X	X	X	X	X	X	X		X	X	X
Adverse event collection (AE)		X	X	X	X	X	X	X	X	X	X	X	X	X	X	X	X	X		X	X	X
Double-blind placebo-controlled challenge		X	X																			
Randomization				X																		
Study drug administration^e^				X	X^j^	X	X^j^	X	X^j^	X^j^		X^j^										
Initial food escalation								X														
Epinephrine auto-injector training^f^								X														
Review OIT action plan^f^								X	X	X	X	X	X	X	X	X	X	X		X		
Up-dosing^g^									X	X	X	X	X	X	X	X	X					X
Food treatment mix dispensation								X	X	X	X	X	X	X	X	X	X					X
Review and dispense of study home dosing diary								X	X	X	X	X	X	X		X	X	X	X	X	X	X

^a^Number of OIT up-dosing visits depends on time needed to reach a final dose of 1500 mg of total proteins that can be tolerated for 2 weeks. Visit W12, W24, W36 and W52 are mandatory even if OIT up-dosing phase has been completed

^b^Followed by monthly weight and height measurements for pediatric patients

^c^Female subject of childbearing potential

^d^At screening, prior medications taken within 6 months prior to V1 will be collected

^e^During the randomized treatment period, investigational product (IP) injections must be separated by at least 11 days. To assess any injection reaction, subject will be monitored at the study site for a minimum of 2 h after first IP injection and then for 30 min after subsequent injection visits. Observation time can be extended at the discretion of the investigator

^f^Can be reviewed any time during the study participation as deemed necessary

^g^Up-dosing will be performed every 2 weeks until maintenance is achieved. Timing of visits is adjusted on treatment tolerance. The OIT dose on up-dosing visits will be determined by the up-dosing rules

^h^Assessment to be performed before DBPCFC and IFE

^i^Assessment to be performed before and after completion of DBPCFC and IFE

^j^For subjects on a biweekly study drug regimen, weighting ≥ 37.5 kg at time of randomisation

^k^Half dosage study drug taper period, 8 mg/kg vs 4 mg/kg vs placebo

^l^Quarter dosage study drug taper period, 4 mg/kg vs 2 mg/kg vs placebo

^m^Procedures during the Unscheduled Visit will be performed as deemed necessary by the Investigator

^n^If maintenance is achieved at W36, a phone call can be performed instead of an onsite visit

^o^Optional

^p^Can be performed on V2 or V3 or anytime prior to V4

^q^Must be performed prior to IFE

*QoL questionnaires include FAQLQ, SF-6Dv2, CHU9D. Questionnaires should be distributed according to the subject age at screening

**Treatment-related cost evaluation questionnaire assesses direct and indirect cost questions. The initial questionnaire includes a sociodemographic and cost assessment section. The follow-up questionnaires only include a cost assessment section. Subjects will be asked to prospectively record the costs associated with the treatment in their diary

### Primary endpoint

Time from initial food escalation (IFE) to target multi-food protein maintenance dose of 1500 mg of total protein (500 mg per food) with study drug.

### Secondary endpoints

1. Change in reactivity threshold to food treatment mix after pre-treatment with study drug.
2. Average up-dosing speed (i.e. percent amount escalated at each visit) while on study drug.
3. Allergic adverse events attributable to food dosing throughout the trial.

### Participant selection

The study will enroll children, adolescent and adults 6 to 25 years old with at least 3 IgE-mediated food allergies who meet all of the inclusion criteria (Table 2) and none of the exclusion criteria (Table 3).

**Table 2:** Inclusion criteria

1. Male or female participants 6 to 25 years old at screening visit
2. History of IgE-mediated allergy to at least three foods within the following list: peanut, milk, egg, wheat, oat, soy, barley, rye, buckwheat, hazelnut, pecan, cashew, pistachio, almond, walnut and sesame
3. Participants currently following a strict avoidance of these three foods
4. Positive SPT with a largest wheal diameter ≥ 6 mm to all three foods
5. Food-specific IgE level greater than 15 kU/L for all three foods
6. Positive DBPCFC to treatment food mix with an eliciting dose ≤ 300 mg of total food protein
7. Signed informed consent and assent

**Table 3:** Exclusion criteria

1. Participants reacting objectively to the placebo during the screening DBPCFC.
2. Severe asthma as defined by GINA 2019 [46]
3. Active or past confirmed eosinophilic oesophagitis
4. Participant currently under allergen immunotherapy
5. Participant/parent with excessive anxiety unlikely to cope with study conditions as per investigator’s opinion
6. Participant/parent unwillingness to comply with study requirements
7. Participant unwillingness to ingest a daily food dose of up to 1500 mg of allergen protein
8. Inability to discontinue anti-histamine medication prior to study procedures
9. Known allergy to omalizumab or its excipients
10. Known allergy to components of the placebo food treatment mix that cannot be substituted without interfering with the blind (i.e.: dates, banana, chocolate syrup)
11. Use of immunosuppression or immunomodulatory drug (including omalizumab) or food oral immunotherapy or investigational treatment or procedure within 1 year
12. Relative contraindication or inability to use epinephrine auto-injector
13. Participants receiving beta-blockers or angiotensin converting-enzyme (ACE) inhibitors
14. Pregnancy or lactation for the duration of the study
15. Any condition that is not compatible with the study treatment or procedures as per investigator judgment

### Screening

First, participants will be screened to determine their eligibility. During screening, a Double-Blind Placebo-Controlled Food Challenge (DBPCFC) will be performed over two separate days. They will ingest increasing amounts of a smoothie containing either placebo or a mix of three of their allergens in an equivalent stoichiometric ratio for their protein content (1:1:1). Participants will be given increasing doses of food protein until a final dose of 300 mg of total food protein (100 mg per food) is administered. Only participants with an objective reaction to the food treatment mix and an eliciting dose of 300 mg or less of total food protein will be admissible.

### Study drug

Once eligibility is confirmed, participant who consent will be randomized using stratified, permuted blocked randomization, using a 2:2:1 allocation. The randomization will be stratified by center and by baseline eliciting dose during DBPCFC (two eliciting dose strata: low eliciting dose (≤ 30 mg of total protein (10 mg per food)) and high eliciting dose [> 30 mg of total protein (10 mg per food)]. They will be randomized to one of the three arm of the study: A: Omalizumab 16 mg/kg per month, B: Omalizumab 8 mg/kg per month or C: Placebo. The online group allocation system will be managed independently by the CHU Ste-Justine Applied Clinical Research Unit.

Participants will be treated with omalizumab at their randomized dosage or placebo for a pre-treatment period of 8 weeks prior to the initial food escalation (IFE). Study drug will be continued for 12 weeks after IFE, for a total of 20 weeks (W-8 to W12). Study drug will be given at full dosage for the first 12 weeks (W-8 to W3) and gradually tapered by reducing the dose by half (50% of initial dosage) from W4 to W7, and again (25% of initial dosage) from W8 to W11 (Fig. 3).

#### Blinding strategy

Because the investigational product (IP) and the placebo are not exactly similar (greater viscosity with active ingredient) and the volume of IP administered may differ depending on treatment assignment, a specific blinding plan was designed.

Briefly, the site pharmacist responsible for the receipt, accountability as well as the reconstitution of IP will remain unblinded. The unblinded pharmacist will have access to the randomization list for his site and will validate all doses automatically calculated by the online randomization system in RedCAP. An unblinded nurse independent from the rest of the team and without any other role in the study will be responsible for administrating the study drug. The dose will be divided in the same number of distinct injections regardless of study arm, but the volume dispensed will be adjusted accordingly (Table 4). The unblinded pharmacist will pre-draw all syringes following a double-verification of the appropriate dose. The unblinded nurse will thus inject the full volume, which will not be documented in the source documents or the subject’s medical chart. Only the number of injections will be documented. This is to avoid participants inadvertently discussing the number of injections in front of blinded personnel who could then deduce their treatment arm.

**Table 4:**
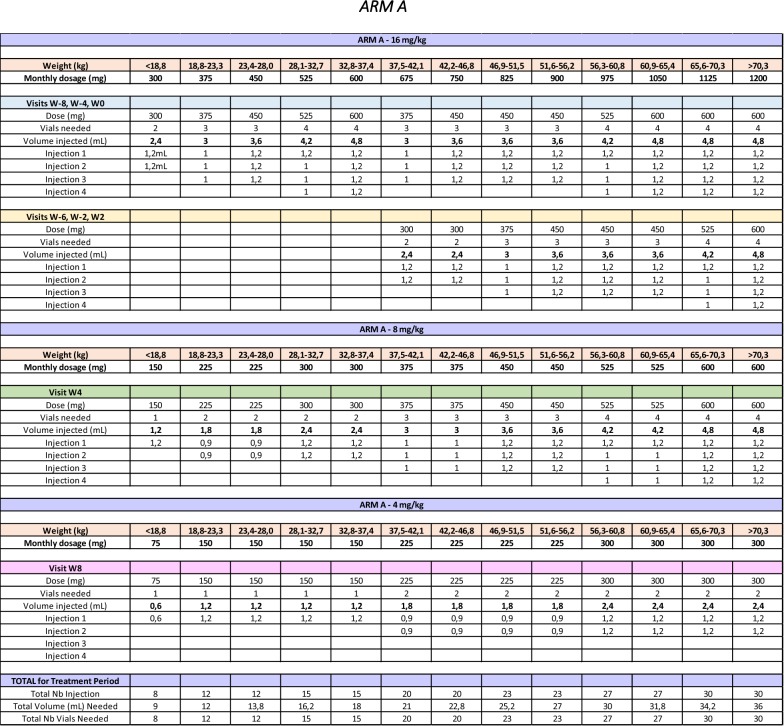

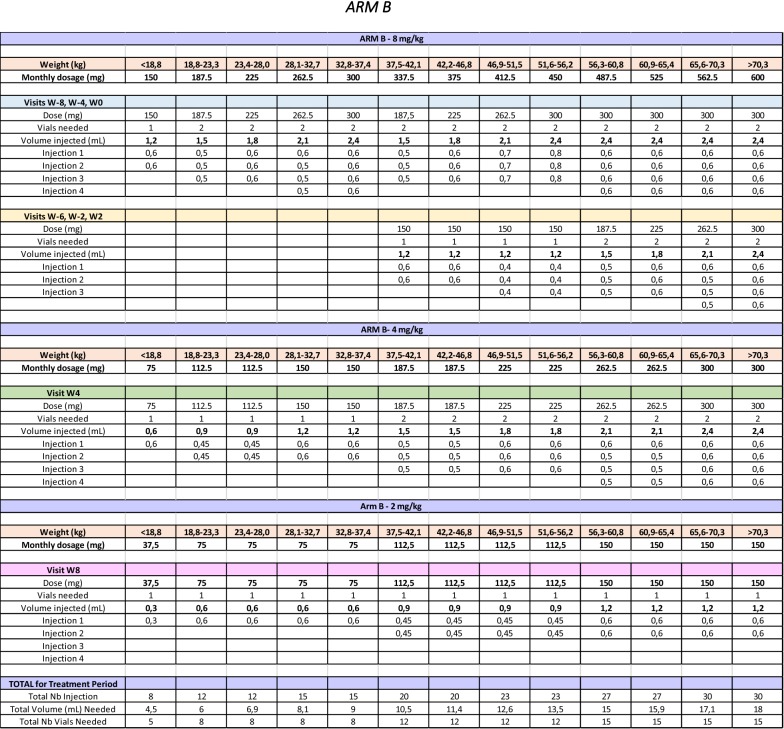

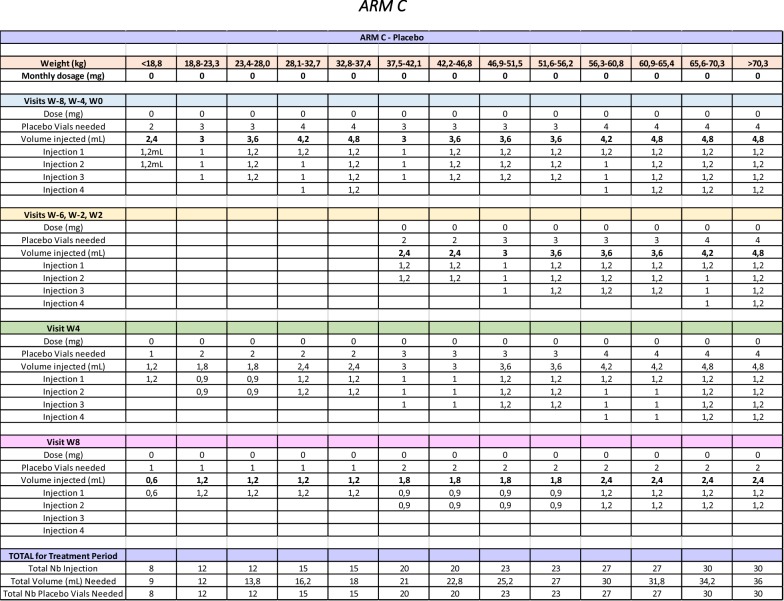
BOOM trial study drug dosage table

Sealed envelopes indicating participant allocation will be kept at the site pharmacy and at the coordinating center, to be used in case of emergency blinding for reasons of safety only.

### Symptom-driven OIT

Multi-food oral immunotherapy will be initiated after the 8-week pre-treatment phase. OIT will be conducted to a mix of three foods from the following list: peanut, milk, egg, wheat, oat, soy, barley, rye, buckwheat, hazelnut, pecan, cashew, pistachio, almond, walnut and sesame.

#### Initial food escalation

At week 8 (day 1), they will undergo the initial food escalation (IFE), which consists in the ingestion of incremental amounts of their food mix every 30 min following the same schedule as the DBPCFC but continuing up to a total of 1500 mg of protein (Table 5) or up to the occurrence of clinically significant symptoms (Table 6).

**Table 5:** Double-blind placebo-controlled food challenge (DBPCFC) and initial food escalation (IFE) schedule

Protein amount in mg (1:1:1)	0.3	1	3	10	30	100	300^a^	600	1050	1500
Observation time (min)	30	30	30	30	30	30	30^b^	30	30	120

^a^Last step for DBPCFC

^b^observed for 2 h during DBPCFC

**Table 6:**
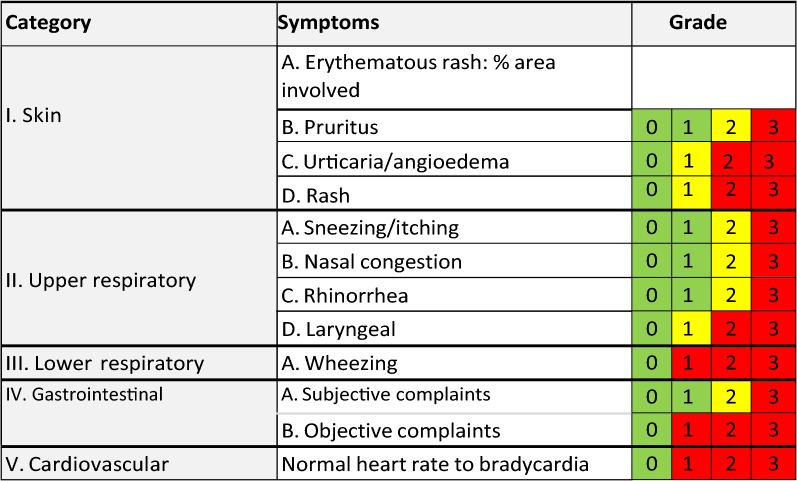
DBPCFC and IFE stopping rules

Stopping rules based on the PRACTALL scoring system. Challenge will be stopped when any symptom reaches the red or when 2 symptoms from different categories reach the yellow. Adapted from Sampson et al. [47]

#### Daily home dosing

Subjects will begin daily home dosing with the highest tolerated dose from IFE. Pre-weighted food dose mix will be dispensed in individual cups and subjects will be instructed to ingest their dose every day around the same time. Participants will be trained on the recognition and appropriate management of dosing reactions and co-factors that increase the likelihood of dosing reactions (infection, peak seasonal allergies, asthma exacerbation, acute stress or fatigue, non-steroidal anti-inflammatory drugs, etc.). In the event of co-factor, the OIT dose will be temporarily decreased by half and the next up-dosing will be postponed until after the co-factor has resolved. The OIT dose will be decreased to the last tolerated dose in the event of systemic or moderate-to-severe local reaction.

#### Up-dosing visits

Participants will return to clinic every other week for a supervised escalation of the food mix amount. To be eligible for up-dosing, the participant must have taken his/her full dose at least 10 times in the last 14 days without any severe local or systemic reaction. On the first up-dosing visit, participants will attempt to double their food amount (+100%). The percent amount escalated on following up-dosing visits will be adjusted based on clinical tolerance to home dosing according to Tables 7 and 8 and until a maintenance dose of 1500 mg of protein (500 mg per food) is reached.

**Table 7:** Symptom-driven up-dosing rules

Symptoms to OIT home dosing since last up-dose	Management
No symptoms at all	Up-dose as planned Double next planned % up-dosing
Transient mild (CoFAR grade 1)	Up-dose as planned Keep next planned % up-dosing the same
Transient moderate (CoFAR grade 2) or persistent mild (CoFAR grade 1)	Up-dose as planned Decrease next planned % up-dosing by half
Persistent moderate symptoms (CoFAR grade 2)	Do not up-dose Decrease next planned % up-dosing by half
At any time, if systemic or severe local reaction (CoFAR grade ≥ 3)	Decrease to previously tolerated dose Decrease next planned % increase by half

**Table 8:** Allergic reaction assessment tool based on the CoFAR grading system

	Grade 1—mild	Grade 2—moderate	Grade 3—severe	Grade 4—life-threatening	Grade 5—death
Intensity	Transient or mild discomfort (˂48 h)	Mild to moderate limitation in activity	Marked limitation in activity	Extreme limitation in activity	
Assistance	No	May be needed	Usually required Parenteral medication(s) are usually indicated	Significant assistance required	
Medical Intervention/Therapy Required	No or minimal	No or minimal	Required	Required	
Hospitalization	No	Possible	Possible	Probable	
May include these symptoms	Pruritus Swelling Rash Abdominal discomfort Other transient symptoms	Persistent hives Wheezing without dyspnea Abdominal discomfort/increased vomiting Other symptoms	Bronchospasm with dyspnea Severe abdominal pain Throat tightness with hoarseness Transient hypotension Other symptoms	Persistent hypotension and/or hypoxia Decreased level of consciousness Associated with collapse and/or incontinence Other life-threatening symptoms	

Participants that react on their escalation will remain on the same dose for another 2 weeks and reattempt up-dosing at half the percent increase of the failed up-dose. If up-dosing fails again, the percent increase will again be decreased by half at each subsequent visit until the up-dose is tolerated.

In the event where the up-dosing rules dictate increasing by a percent amount that was previously failed, then the participant must repeat one additional uneventful visit increasing with the current percentage before proceeding to this new percent increase.

#### Maintenance

Up-dosing visits will take place following the above-mentioned rules until a maintenance dose of 1500 mg is reached. The subject will remain on that daily dosage for at least 2 weeks after which they will transition to food equivalents. Participants will remain on that maintenance dose until at least 12 months after the IFE.

### Concomitant medication

In clinic and at home, acute reactions to DBPCFC or OIT food doses will be treated according to WAO Anaphylaxis Guidelines [38], as deemed appropriate by the investigator. Medication can be prescribed to participants to prevent symptoms related to OIT as in real-life. The indication and choice of prophylactic medication is determined by the investigator. Type 1 and 2 anti-histamines, leukotriene receptor antagonists, proton-pump inhibitors, prostaglandin E1 analogs, mast cell stabilizers or swallowed corticosteroids can be used depending on the situation. Their use will be documented in the patient diary and concomitant medication log. The decision to perform endoscopy/biopsy remains at the investigator’s discretion and should be balanced with the risk of delaying proper treatment. As a reference, the Canadian clinical practice guidelines on OIT recommend that endoscopy and biopsy be used to confirm the diagnosis in suspected cases not responding to dose adjustments or medication [39].

### Follow-up of participants

Participants will be followed for a minimum of 12 months after the initial food escalation. Follow-up of participants will end when the last randomized participant has reached 12-month follow-up. Participants discontinuing treatment for any reason will be offered follow-up care and invited to complete an early termination visit to collect all data that would have otherwise been collected at the end of study (Table 1).

### Assessment of efficacy

The primary endpoint is defined as the first visit at which an attempt to escalate to 1500 mg of protein of treatment food mix is successful (e.g. the lack of any systemic or of local reaction requiring treatment). This will be reaffirmed on the following visit by confirmation that the dose was successfully maintained at home in the following 2 weeks. The main secondary outcomes will be measured as follows. Extent of pharmacologic desensitization from omalizumab pre-treatment will be measured by comparing the amount of food required to elicit clinically significant symptoms in the IFE compared to baseline DBPCFC (Table 5). Both procedures use the same objective stopping criteria to ensure comparability. Up-dosing speed will be measured as the average of log percent food increase on up-dosing visits between D1 and W12, adjusted for the number of days between visits. Mean cumulative function of allergic adverse events attributable to food dosing will be captured using a daily dosing diary throughout the trial, including during maintenance. The 3-month wash-out period following discontinuation of omalizumab will be of special interest with regards to continued dose tolerance. Allergic events occurring during escalation visits will be documented directly on case report forms. All moderate to severe reactions (CoFAR grade 2 or higher) occurring at home or during up-dosing visits will also be reviewed and documented in the AE log and on the OIT-reaction report form.

### Safety variables

All adverse events (AEs) and severe adverse events (SAEs) occurring during the study, including intercurrent illnesses, will be documented in the e-CRF. Reactions attributed to food dosing during OIT will be treated as AEs of special interest (AESI) since they are also measures of treatment efficacy as described above. The following AEs will also be considered AESI given prior reports in relation to omalizumab: arterial thromboembolic events, malignant neoplasms, anaphylaxis/anaphylactoid reactions not attributable to ingestion of food allergen.

**Fig. 4:**
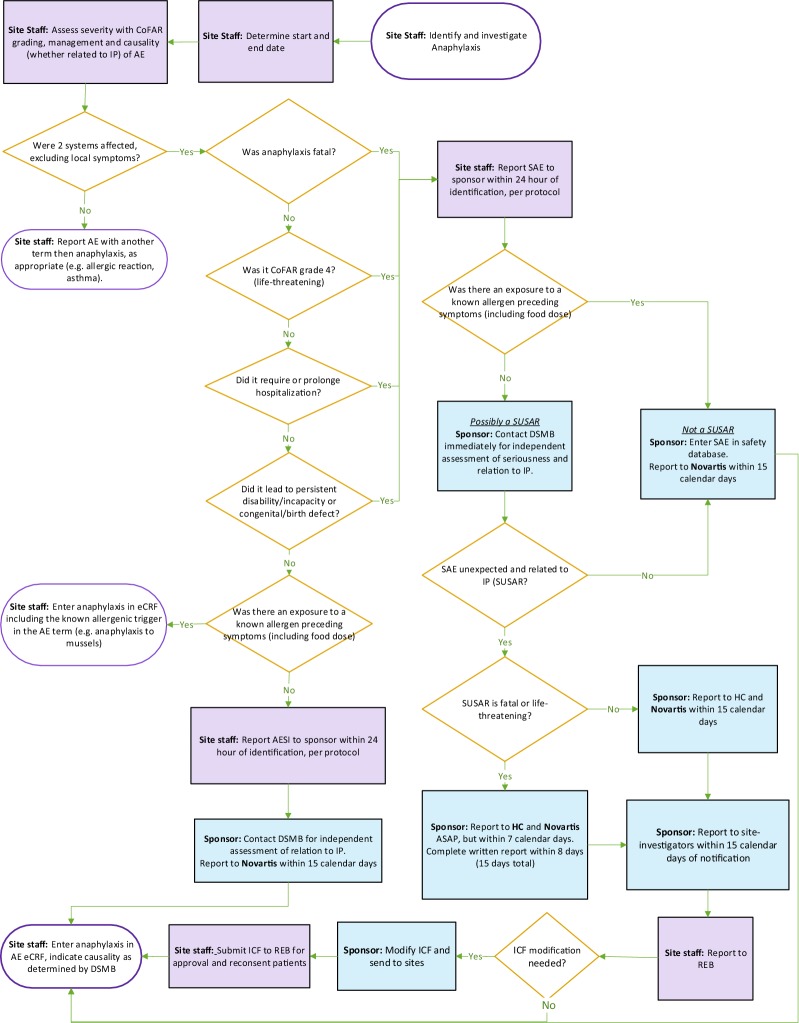
Anaphylaxis reporting plan. SUSAR: suspected unexpected serious adverse reaction; ICF: informed consent form; IP: investigational product; AESI: adverse event of special interest, DSMB: Data Safety Monitoring Board; AE: adverse event; HC: Health Canada; REB: Research Ethics Board

The proper reporting of anaphylaxis in this trial poses a specific challenge considering it is an expected side effect of both OIT and omalizumab. A specific anaphylaxis reporting procedure was developed to ensure proper reporting of anaphylaxis causality for drugs studied in the context of food OIT (Fig. 4).

### Statistical methods

The primary outcome for the trial will be time-to-maintenance dose which will be compared between groups using the stratified log-rank-analysis (stratified by center and baseline eliciting dose during DBPCFC). Each of the three pairwise comparison (omalizumab 16 mg/kg vs omalizumab 8 mg/kg, omalizumab 8 mg/kg vs placebo, omalizumab 16 mg/kg vs placebo) will be evaluated using the Bonferroni correction for multiple tests.

Data will be analyzed using the intent-to-treat approach. In the log-rank analysis, this means that patients who drop-out or are lost to follow-up for any reason will be considered as having never reached the outcome.

To control for the risk of multiple outcome measures, secondary analyses will only be performed if at least one of the active arm is found better than the placebo arm in the primary analysis. Comparison of treatment efficacy based on sex is also planned as an exploratory objective.

### Sample size calculation

The sample size will be primarily driven by the 16 vs 8 mg/kg comparison. Assuming a median time-to-maintenance of 2 weeks in the 16 mg/kg arm based our clinical cohort, a sample of 72 participants (36 in each arm) would confer a power of 0.80 to detect a HR = 2.2 of time-to-maintenance with an alpha of 0.017 (considering 3-way testing between the study arms), assuming administrative censoring at 52 weeks. A HR of 2.15 would mean 2.3 additional OIT weeks, which was considered the minimal clinically relevant difference (i.e. one up-dosing visit).

Assuming a median time-to-maintenance of 6 weeks in the 8 mg/kg arm, a sample of 54 participants (18 in placebo arm) would confer a power of 0.80 to detect a HR = 2.54 if a time-to-maintenance with an alpha of 0.017, assuming administrative censoring at 52 weeks. A HR of 2.54 would mean 9.2 additional OIT weeks, which was considered the minimal clinically relevant difference to consider adding adjunct drug therapy.

### Quality assurance

The Sponsor will conduct a site visit to verify the qualification of each Investigator, inspect the site facilities, and inform the Investigator of her/his responsibilities and the required procedures for ensuring adequate site selection and correct documentation.

All data generated by the site personnel will be immediately captured electronically at each study center using e-CRFs, with a maximum delay of 3 days. Data from external sources (such as laboratory data) will be imported into the database. Computerized edit-checks will be developed in addition to manual review to detect any discrepancies and to ensure consistency of the data. An electronic audit trail system will be used to track all data changes in the database subsequent to the first data entry.

During the study, a site monitor will conduct site visits to review protocol compliance, compare e-CRF entries and individual subject’s medical records, assess drug accountability, and ensure that the study is being conducted according to ethical and pertinent regulatory requirements. The e-CRF entries will be verified with source documentation. The review of medical records will be performed in a manner to ensure that subject confidentiality is maintained. Site monitoring visits will begin within 2 weeks of the first randomized subject and are planned every 12 weeks thereafter, based on site activity. In addition, the Sponsor may conduct audits at the investigative sites including, but not limited to, drug supply, presence of required documents, the informed consent process, respect of GCP standards and comparison of e-CRFs with source documents.

A Data Safety Monitoring Board (DSMB) composed of 3 independent members and chaired by a clinical trialist with experience in running multi-center food allergy trials will have access to safety reports every 6 months and will make appropriate recommendations to the trial steering committee (composed of the principal investigators from each site, the study coordinator and the study methodologist). There are no interim analysis planned for efficacy outcomes.

Internal audits of the coordinating center (CHU Sainte-Justine) are also planned by the institution pharmaceutical research quality assurance committee to ensure compliance. In addition, external audits by Health Canada could be conducted at any moment during or after the study.

### Dissemination

Results of the study will be communicated at scientific conferences and journals. These will be written by the investigators without any restriction or recourse to professional writers.

## Discussion

The BOOM study is a multi-center randomized clinical trial that uses an innovative design with a pragmatic OIT up-dosing system to compare different dosages of omalizumab used as adjunct to safely accelerate oral immunotherapy. Contrarily to more classic designs based on fixed up-dosing schedules, this approach allows to differentiate the extent to which differences in up-dosing phase duration can be attributed to the presence of omalizumab rather than to the up-dosing schedules themselves.

To ensure safety, the symptom-driven up-dosing rules were first piloted and successfully implemented at our OIT clinic at Sainte-Justine Hospital. Following implementation, there were actually less systemic reactions to up-dosing (6 ± 5 systemic reactions per 1000 up-dosing visits) compared to before when using fixed schedules (with 15 ± 7 per 1000 with fixed schedules). One should be careful not to over-interpret this observation, which is based on historical comparison. However, it does provide strong argument supporting the safety of the approach. Importantly, the adaptive system allowed some patients without omalizumab to progress at a much faster pace than what would have been allowed with the traditional fixed schedule, demonstrating the risk of bias if the study had been designed to compare different fixed schedules.

Because the criteria used for determining up-dosing amount is tolerance to the food dose, progression with up-dosing should directly reflect the impact of the study drug on OIT safety and tolerability. In a certain way, this makes the primary outcome of time-to-maintenance a compound of both the safety and efficacy gain with omalizumab. It is also potentially the most relevant outcome from a payer’s perspective as each visit saved compensates part of the cost of medication. It is important to also consider indirect costs, which are especially relevant in cases where patients need to travel long distances, sometimes by plane, to come to their up-dosing appointments. Indirect costs will be captured by patient questionnaire throughout the study.

Another important advantage of the adaptive up-dosing system is that it allows to maintain blinding of study arm when comparing standard OIT to omalizumab-enabled accelerated OIT, which would have been impossible with fixed schedule. If those had been used, the difference in dose progression would have allowed participants and investigators to rapidly identify the study arm, even with perfect masking of study drug/placebo. To solve this issue, previous studies have used either the standard or the accelerated schedule in all participants [36]. However, as mentioned above, while this helps demonstrate that patients are unlikely to successfully the accelerated schedule without omalizumab, because it does not allow to individualize the speed it prevents the quantification the benefit attributable to omalizumab (i.e. how much slower did the schedule need to be for patients on placebo to have tolerated it).

An important way to decrease cost is by rationalizing the use of expensive medication. This trial will be the first to address the question of optimal dosage for omalizumab in the context of OIT. A previous trial with TNX-901, another anti-IgE monoclonal, and our own retrospective cohort analysis [40] both indicate that the effect of pre-treatment with omalizumab on reactivity threshold follows a linear relationship with dosage per weight, independent of IgE, hence the dosage strategy based on weight, irrespective of total IgE used in this trial [17, 40]. The primary outcome of time-to-maintenance is dependent on this increase in reactivity threshold, which allows a higher starting dose, but also on OIT progression during following up-dosing appointments. The extent to which omalizumab plays a role in increasing up-dosing speed past the initial escalation has not been established and will be assessed as a secondary objective in this trial. This will serve to inform on the optimal duration of co-treatment with omalizumab after the IFE, which is presently unknown.

The 16 mg/kg monthly dosage corresponds to the highest dose for which there is published safety data (600 mg q 2 weeks in adults) and which has previously been approved for asthma in Europe [41]. Because the dose-related efficacy of omalizumab in our previous retrospective cohort on oral immunotherapy was shown to follow a logarithmic scale [40], a second dosage was chosen at half the maximum dosage, at 8 mg/kg.

Here, a co-treatment phase of 12 weeks following the IFE was chosen based on previous protocols and also to allow time for a progressive tapering of the study drug. We and other groups have highlighted an increased rate of adverse events during the months following omalizumab discontinuation and there are reports of failure to wean patients from omalizumab, mostly due to new onset or return of previously controlled gastro-intestinal symptoms from the food dose [19, 34, 40, 42, 43]. Because protective omalizumab-IgE complexes can sometimes persist for more than 6 months, we ensured to provide a sufficient follow-up period to capture these symptoms. The efficacy of the tapering strategy to ease the transition as well as the optimal duration of co-treatment phase will likely warrant a specific trial of their own.

Another novel feature in this trial is the recourse to a DBPCFC to the food treatment mix. In previous trials on multi-food OIT, DBPCFC were conducted individually for each food which makes for challenging interpretation of study data [37]. It also significantly increases costs for study sites since DBPCFC is one of the most expensive research procedures in food allergy. Finally, it creates a barrier to recruitment, as participants can be reluctant to undergo multiple screening DBPCFCs, for fear of reaction but also in terms of family logistics since it involves multiple day-long visits. The idea here was to extend the concept of the OIT food mix being an inseparable whole from treatment to outcome measures. This means that discrete DBPCFC data for individual allergen will be lost in exchange for a clearer, more generalizable outcome assessment.

The Canadian clinical practice guidelines on OIT recommend that the final target dose for the therapy should be guided by the patient’s individual clinical response and personal goals [39]. Here, given the research context which requires more standardization, the target multi-food protein maintenance dose was established at 500 mg protein per allergen, for a total of 1500 mg of allergen protein. This amount was chosen as a compromise offering a reasonable and easily measurable target maintenance dose for all 16 allergens once converted to equivalent food forms. For comparison, the target dose of the PALISADE trial used a target maintenance dose of 300 mg of peanut protein [44, 45], whereas target doses of 800 mg and 2000 mg were used in the STOPII [2] and the PROTECT trials [36], respectively.

Finally, the BOOM trial will provide data that is directly complementary to another ongoing multi-center study addressing the use of omalizumab in food allergy, currently recruiting in the US. The OUtMATCH trial (NCT03881696) is based on a 2-part design. In the first part, it will compare omalizumab used as monotherapy to placebo in subjects with at least 3 food allergies. The primary outcome measure will be change in reaction threshold measured on DBPCFC to the food mix. In its second part, subjects will be randomized to either remain on omalizumab continuously or proceed with an accelerated OIT to their 3-food mix and then discontinue omalizumab, in a double-blinded manner. This will allow comparison of omalizumab-enabled accelerated OIT to yet another therapeutic option (continuous monotherapy with omalizumab).

## Conclusion

In conclusion, the unique design of the BOOM trial will allow to yield critical data toward the use of omalizumab as adjunct to accelerated OIT, notably in regards to optimal dosage and superiority compared to slower OIT without omalizumab. Together with the OUtMATCH trial, it will prove critical in defining the parameters for the clinical use of omalizumab in food allergy.

## Data Availability

Final trial dataset and complete protocol will be shared by the investigator-sponsor at time of publication.

## Abbreviations

AE: Adverse event
AESI: Adverse event of special interest
BOOM: Double-Blind, randomized controlled trial comparing two dosages of Omalizumab to placebo to accelerate a symptom-driven Oral immunotherapy schedule for the treatment of Multiple food allergies
DBPCFC: Double-Blinded Placebo-Controlled Food Challenge
HR: Hazard ratio
IFE: Initial food escalation
IgE: Immunoglobulin E
IP: Investigational product
OEAOIT: Omalizumab-enabled accelerated oral immunotherapy
OIT: Oral immunotherapy
RR: Relative risk
SAE: Serious adverse event
