# Correction to: Protocol for a double-blind, randomized controlled trial on the dose-related efficacy of omalizumab in multi-food oral immunotherapy

**DOI:** 10.1186/s13223-020-00435-z

**Published:** 2020-05-20

**Authors:** Alexandra Langlois, Marie-Hélène Lavergne, Hélène Leroux, Kerstin Killer, Pauline Azzano, Louis Paradis, Kathryn Samaan, Jonathan Lacombe-Barrios, Thomas Eiwegger, Julia Upton, Gordon Sussman, Thomas Poder, Benoît Mâsse, Anne Des Roches, Philippe Bégin

**Affiliations:** 1 Department of Allergy and Immunology, Centre Hospitalier Universitaire Sainte-Justine, Montreal, QC Canada; 2 Centre Hospitalier Sainte-Justine Research Center, Montreal, QC Canada; 3 Department of Allergy and Immunology, Centre Hospitalier de l’Université de Montréal, 3175 Chemin de la Côte Sainte-Catherine, Montreal, QC H3T1C5 Canada; 4 Division of Immunology and Allergy, Department of Paediatrics, The Hospital for Sick Children, The University of Toronto, Toronto, ON Canada; 5 Gordon Sussman Clinical Research, Toronto, ON Canada; 6 Department of Management, Evaluation and Health Policy, School of Public Health, Université de Montréal, Montreal, QC Canada; 7 Research Center of the Institut Universitaire de Santé Mentale de Montréal, Montreal, QC Canada; 8 School of Public Health, Université de Montréal, Montreal, QC Canada

## Correction to: Allergy Asthma Clin Immunol (2020) 16:25 10.1186/s13223-020-00419-z

The names of Thomas Eiwegger, Julia Upton, Gordon Sussman and Thomas Poder were inadvertently omitted from the authorship of the original article [1]. Thomas Eiwegger, Julia Upton, Gordon Sussman and Thomas Poder had participated in meetings and exchanges regarding the trial design and their names are added to recognise their contribution to the conception of the protocol.

The full and correct authorship has been updated in the original article and included in the author list of this Correction article.

The ‘Competing interests’ section is also corrected here to read: The authors declare no conflict of interest related to the study. KS reports personal fees from Novartis outside the submitted work. TE reports grants from DBV technologies, Innovation fund Denmark, CIHR, and Regeneron and personal fees from ALK ourside the submitted work. He serves as associate editor for Allergy. JU reports personal fees from Food Allergy Canada, ALK-Abelló, Kaleo, and grants from Toronto SickKids Foundation, DBV technologies, Regeneron, and ALK-Abelló outside the submitted work. GS reports personal fees from Novartis, Aralez, CSL Behring, Sanofi, Pediapharm, GSK, Genentech, DBV technologies, Aimmune, Astrazeneca, Stallergenes, Merck, Pfizer, Dyax, Biocryst, Greencross, Kendrion, Shire, Leopharma, Regeneron, mdBriefCase and grants from Novartis, GSK, Genentech, DBV technologies, Aimmune, CSL Behring, Astrazeneca, Stallergenes, Merck, Pfizer, Dyax, Biocryst, Greencross, Kendrion, Leo Pharma, Regeneron, Sanofi, Blueprint, ALK, Amgen, Cliantha outside the submitted work. ADR reports grants from Merk and ALK outside the submitted work. PB reports personal fees from Novartis, Pfizer, Sanofi, ALK and Aralez, as well as grants from DBV technologies, Regeneron and Sanofi outside the submitted work.
